# Identification of serum glycobiomarkers for Hepatocellular Carcinoma using lectin microarrays

**DOI:** 10.3389/fimmu.2022.973993

**Published:** 2022-10-20

**Authors:** Yue Zhang, Sihua Zhang, Jianhua Liu, Yunli Zhang, Yanjie Liu, Shuang Shen, Fangfang Tian, Gaobo Yan, Yongqing Gao, Xiaosong Qin

**Affiliations:** ^1^ Department of Laboratory Medicine, Shengjing Hospital of China Medical University, Shenyang, China; ^2^ Liaoning Clinical Research Center for Laboratory Medicine, Shenyang, China; ^3^ Department of Laboratory Medicine, The First Affiliated Hospital of Jinzhou Medical University, Jinzhou, China; ^4^ Department of Laboratory Medicine, Chaoyang Central Hospital, Chaoyang, China; ^5^ Department of Laboratory Medicine, Huludao Central Hospital, Huludao, China; ^6^ Department of Laboratory Medicine, Fuxin Central Hospital, Fuxin, China; ^7^ Department of Laboratory Medicine, Dandong Central Hospital, Dandong, China; ^8^ Department of Laboratory Medicine, Tieling Central Hospital, Tieling, China

**Keywords:** Hepatocellular carcinoma, lectin microarray, glycosylation, immunoglobulin G, immunoglobulin M, biomarker

## Abstract

**Objective:**

Hepatocellular carcinoma (HCC) is the sixth most commonly occurring cancer and ranks third in mortality among all malignant tumors; as a result, HCC represents a major human health issue. Although aberrant glycosylation is clearly implicated in HCC, changes in serum immunoglobulin (Ig)G and IgM glycosylation have not been comprehensively characterized. In this study, we used lectin microarrays to evaluate differences in serum IgG and IgM glycosylation among patients with HCC, hepatitis B cirrhosis (HBC), or chronic hepatitis B (CHB), and healthy normal controls (NC) and aimed to establish a model to improve the diagnostic accuracy of HCC.

**Methods:**

In total, 207 serum samples collected in 2019–2020 were used for lectin microarray analyses, including 97 cases of HCC, 50 cases of HBC, 30 cases of CHB, and 30 cases of NC. Samples were randomly divided into training and validation groups at a 2:1 ratio. Training group data were used to investigate the diagnostic value of the relative signal intensity for the lectin probe combined with alpha-fetoprotein (AFP). The efficacy of models for HCC diagnosis were analyzed by receiver operating characteristic (ROC) curves.

**Results:**

In terms of IgG, a model combining three lectins and AFP had good diagnostic accuracy for HCC. The area under the ROC curve was 0.96 (P < 0.05), the sensitivity was 82.54%, and the specificity was 100%. In terms of IgM, a model including one lectin combined with AFP had an area under the curve of 0.90 (P < 0.05), sensitivity of 75.41%, and specificity of 100%.

**Conclusion:**

Estimation of serum IgG and IgM glycosylation could act as complementary techniques to improve diagnosis and shed light on the occurrence and development of the HCC

## Introduction

Glycosylation is one of the most common protein post-translational modifications; more than half of all human proteins are estimated to be glycosylated with different glycan chains (1). Glycosylation is involved in many critical biological processes, such as cell growth (2), protein folding and conformational stability (3), and differentiation (4). Many studies have shown that aberrant glycosylation plays essential roles in a variety of diseases, from cancer to infectious diseases and immune-related diseases (5). Glycosylation is fairly common in tumor cells, and the complexity of glycosylation increases the functional diversity of tumor molecules (6). For instance, immunoglobulin (Ig)G is crucial in activating the complement systems, and its galactosylation (a form of glycosylation) is altered in a variety of cancers (7).

Hepatocellular carcinoma (HCC) is the sixth most commonly occurring cancer. Its mortality rate ranks third among all malignant tumors (8). Nearly 80% of HCC cases are related to chronic hepatitis B (HBV) or hepatitis C virus (HCV) infection (9). Researchers have found that a quarter of people infected with HBV in childhood will progress to cirrhosis or even HCC (10, 11). The liver synthesizes the majority of glycosylated serum proteins, and changes in protein glycosylation are essential for the pathogenesis and progression of major liver diseases (12, 13). Glycosylation of major membrane receptors can influence tumor cell adhesion, motility, and invasiveness (14). In fact, glycosylation is believed to be altered in the hepatocytes undergoing deterioration due to HCC.

Immunoglobulin has largely been neglected as it is not produced by hepatocytes and lacks structural complexity; however, serum immunoglobulin accounts for 50% of serum glycoproteins. Moreover, IgG glycosylation at Asn297 of the CH2 domain (CH2Asn-297) within the Fc region is essential for maintaining the spatial configuration and stability of IgG (15). IgG secretion has been detected in tumor cells, and the inhibition of IgG secretion can inhibit the growth of tumor cells (16, 17). Studies have reported IgG glycosylation in other cancers, such as lung and ovarian cancer (18, 19). However, changes in serum IgG and IgM glycosylation in patients with HCC have not been determined.

Alpha-fetoprotein (AFP) is the most common serum biomarker used to detect HCC (20). Unfortunately, not all patients with HCC have elevated serum AFP, which is observed in 60–70% of the patients (21). Some studies have shown that lectin-reactive AFP (AFP-L3) and des-gamma-carboxy prothrombin (DCP) are more effective markers than AFP alone (22–24). However, the sensitivity of AFP-L3 is only 55%, and its applicability in diagnosing HCC is suboptimal (25). Therefore, non-invasive and sensitive markers are urgently needed for HCC diagnosis.

In this study, we used lectin microarrays to evaluate differences in serum IgG and IgM glycosylation in patients with HCC, hepatitis B cirrhosis (HBC), chronic hepatitis B (CHB), and healthy normal controls (NC), and established a combined diagnostic model to improve the accuracy of HCC detection. We found that serum IgG and IgM glycosylation tests could complement existing *in vitro* techniques and improve diagnosis and these glycosylation process may be involved in the occurrence and development of HCC.

## Materials and methods

### Patients

The lectin microarray was used to evaluate 207 serum samples collected between 2019 and 2020, including 97 cases of HCC, 50 cases of HBC, 30 cases of CHB, and 30 cases of NC (**Table 1**). The rank sum test was used to compare signal values. At a ratio of 2:1, 207 samples were randomly divided into a training group (138 cases, including 65 HCC, 20 CHB, 33 HBC, and 20 NC) and validation group (69 cases, including 32 HCC, 10 CHB, 17 HBC, and 10 NC). For HCC group, serum samples were collected from hospitalized patients with HBV-associated primary HCC. Patients with HCC with HCC were diagnosed with primary HCC by pathology after percutaneous liver aspiration biopsy or surgery, had a history of hepatitis B,and had not used hepatotoxic drugs within the past year. Exclusion of HCC due to the progression of hepatitis C, alcoholic liver disease, and autoimmune liver disease. General demographic information, including the sex ratio and average age of patients, corresponding to serum samples in the two groups were matched to the extent possible, and there were no significant differences in these parameters between the two groups. The patient characteristics are shown in **Table 1**. Serum samples were collected from the patients using gel and clot activator tubes during morning fasting. After 30 min, the tubes were centrifuged at 3500 rpm for 10 min. The serum samples were transferred to corresponding EP tubes and stored at –80°C until use.

**Table 1:** Clinical characteristics of patients.

		NC	CHB	HBC	HCC	P
n		30	30	50	97	
Male (%)		21 (70.0)	21 (70.0)	35 (70.0)	70 (72.2)	0.99
Age		54.7 ± 11.3	52.5 ± 9.4	55.0 ± 10.6	56.8 ± 9.8	0.209
AST (U/L)		19.7 ± 4.6	41.6 ± 59.5	55.1 ± 149.5	51.6 ± 75.1	0.35
ALT (U/L)		17.8 ± 5.6	58.2 ± 108.0	48.5 ± 96.7	41.1 ± 5.1	0.187
GGT (U/L)		19.3 ± 9.4	29.0 ± 23.2	45.9 ± 55.3	66.6 ± 80.3	0.001
BilT (umol/L)		12.3 ± 2.0	13.5 ± 5.1	26.6 ± 20.6	17.0 ± 12.2	< 0.001
BilD (umol/L)		3.2 ± 0.8	4.0 ± 1.4	10.5 ± 12.0	7.2 ± 8.8	0.001
ALB (g/L)		45.5 ± 1.8	44.2 ± 3.7	37.6 ± 8.2	41.1 ± 5.1	< 0.001
TP (g/L)		73.6 ± 2.8	71.5 ± 5.2	66.1 ± 8.5	70.4 ± 7.2	< 0.001
lgG (g/L)		12.4 ± 2.26	12.2 ± 3.0	13.3 ± 3.7	12.0 ± 4.3	0.29
lgM (g/L)		0.9 ± 0.4	1.1 ± 0.7	1.0 ± 0.5	1.0 ± 0.7	0.665
AFP (g/L)		3.7 (2.6-5.7)	2.5 (2.0-4.1)	3.3 (2.2-6.1)	34.8 (5.1-379.7)	0.053
PIIIP (ng/mL)			18.9 ± 8.3	47.8 ± 59.7		0.017
HA (ng/mL)			70.0 ± 37.3	221.5 ± 374.5		0.033
LN (ng/mL)			7.8 ± 5.5	37.9 ± 58.2		0.011
CIV (ng/mL)			17.5 ± 6.9	49.8 ± 63.2		0.012
CEA (ng/mL)					2.6 ± 1.6	
CA199 (U/mL)					21.42 ± 19.0	
TNM	I-II				85 (87.6)	
	III-IV				12 (12.4)	
Tumor size	≤5cm				73 (75.3)	
	>5cm				24 (24.7)	
Tumor number	Single				75 (77.3)	
	Multiple				22 (22.7)	

### Lectin microarray

A commercial lectin microarray (purchased from BCBIO Biotech, Guangdong, China)containing 56 lectins was used to investigate the glycopatterns of serum IgG and IgM. Details of the 56 lectins are listed in **Supplementary Table S1**.The lectin microarrays were removed from –80°C storage and placed at room temperature for 30 min. Then, the lectin microarrays were incubated at room temperature for 3 h with blocking buffer [(3% bovine serum albumin in phosphate-buffered saline (PBS)]. After washing and drying, 200 µL of sample (1:500 dilution) was applied to the microarray and incubated overnight at 4°C. After washing three times with PBS, the microarrays were incubated with 3 mL of mouse serum for 1 h. The microarrays were washed three times with PBS and twice with deionized water, followed by incubation with 3 mL of secondary antibody for 1 h. After three washes with PBS and two washes with deionized water again, the microarrays were dried and scanned using the GenePix 4000B Microarray Scanner at wavelengths of 532 nm and 635 nm.

### Statistical analysis

All statistical analyses were performed using SPSS 25.0 and plots were drawn using GraphPad Prism 7. P < 0.05 was considered statistically significant. The following thresholds were set for differential lectin identification: fold change ≥ 1.5 and P < 0.05.

## Results

### Distinct glycosylation patterns between HBC, CHB, NC, and HCC

Serum samples of 97 patients with HCC, 50 patients with HBC, 30 patients with CHB, and 30 NC were detected by lectin microarrays. The mean age of the HCC group (n= 97) was 56.8 years, and 72.2% was male; the mean age of the HBC (n= 50) was 55.0 years, and70% was male; the mean age of CHB (n= 30) was52.5years, and70% was male; and the mean age of NC (n= 30) was54.7years, and70% was male. General demographic information, including the sex ratio and average age of patients, corresponding to serum samples in the two groups were matched to the extent possible, and there were no significant differences in these parameters between the two groups. Each group had no significant difference in AST, ALT, IgG, and IgM.

**Figure 1:**
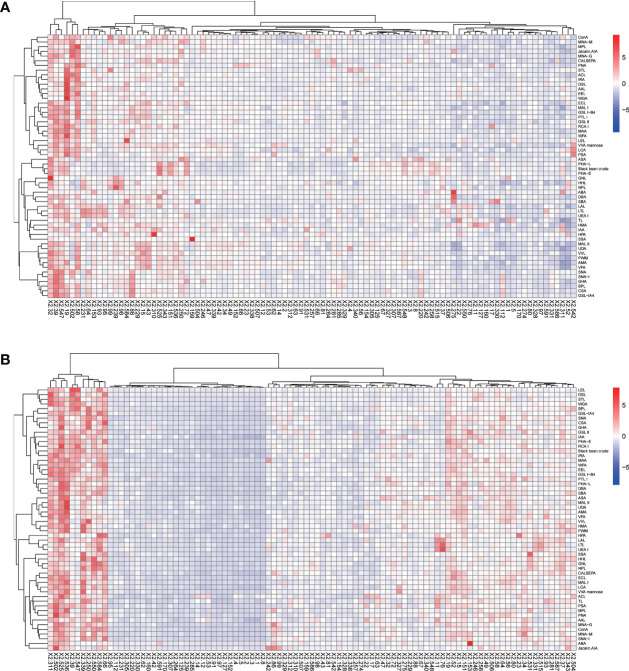
Heatmap of lectins in patients with HCC. **(A)** Lectins of serum IgG. **(B)** Lectins of serum IgM. Red indicates overexpression and blue underexpression. The columns correspond to the 97 HCC samples.

**Figure 2:**
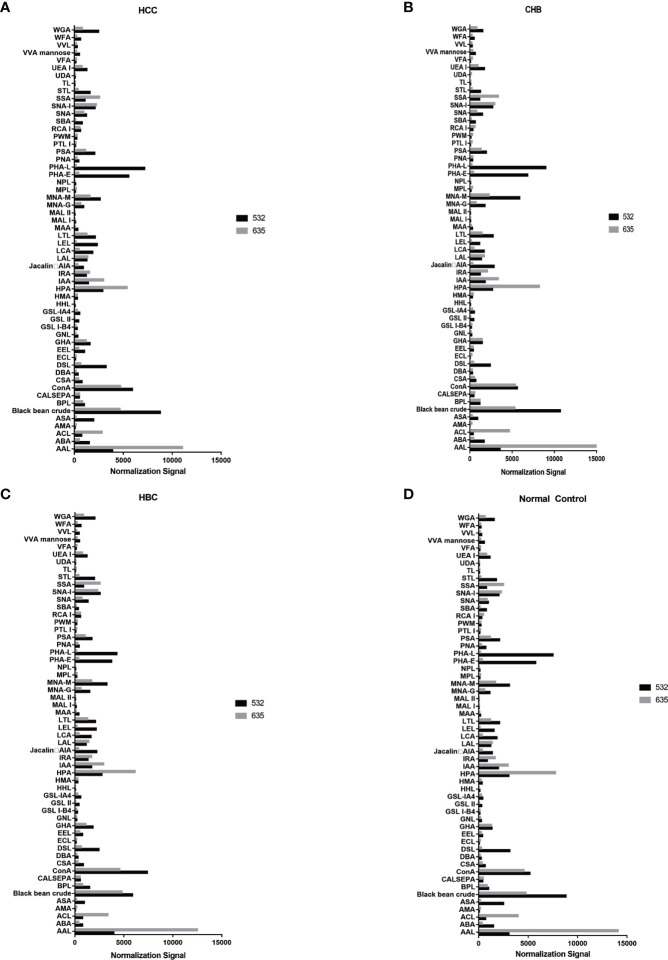
Distribution of normalized signal intensity values for 56 lectins. **(A)** Normalized values of 56 lectins in HCC. **(B)** Normalized values of 56 lectins in CHB. **(C)** Normalized values of 56 lectins in HBC. **(D)** Normalized values of 56 lectins of NC. 532 represents IgG, 635 represents IgM.

The positive signal intensities in the HCC group were mainly detected in lectins, e.g., PHA-L, PHA-E, DSL, ConA, and black bean crude (**Figures 1**, **2**).

### Serum IgG glycosylation determined by a lectin microarray analysis

**Figure 3:**
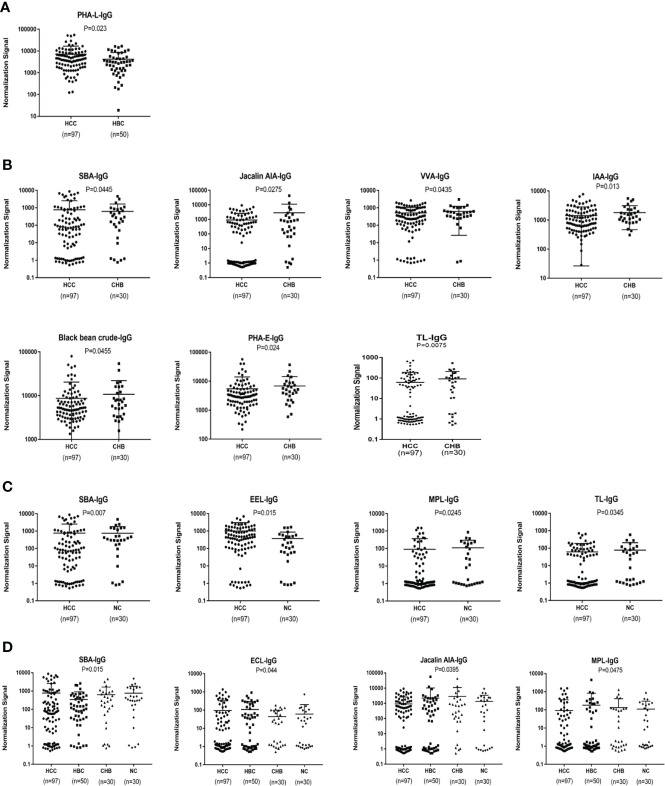
Specific lectins of serum IgG detected by a lectin microarray. **(A)** Specific differences in serum IgG glycosylation between HCC and HBC. **(B)** Specific differences in serum IgG glycosylation between HCC and CHB. **(C)** Specific differences in serum IgG glycosylation between HCC and NC. **(D)** Specific differences in serum IgG glycosylation among HCC, CHB, HBC, and NC.

An analysis of serum IgG glycosylation showed that PHA-L signal intensities differ between HBC and HCC (P < 0.05)(**Figure 3A**). Seven of 56 lectins showed differences in signal intensities between the CHB and HCC groups (P < 0.05); SBA had a higher signal intensity and the signal intensities of AIA, VVA, IAA, Black bean crude, PHA-E, and TL were significantly lower (P < 0.05) (**Figure 3B**). Compared with intensities in NC, SBA and EEL signal intensities were higher, while MPL and TL signal intensities were lower in patients with HCC (P < 0.05) (**Figure 3C**). HBC, CHB, and NC were taken as the control group for subsequent analyses. Two differential lectins (SBA and ECL) with high IgG glycosylation levels in the HCC group were identified (P < 0.05), while AIA and MPL were identified as significantly differential lectins with low IgG glycosylation levels in the HCC group (P < 0.05) (**Figure 3D**).

### Serum IgM glycosylation patterns determined by a lectin microarray analysis

**Figure 4:**
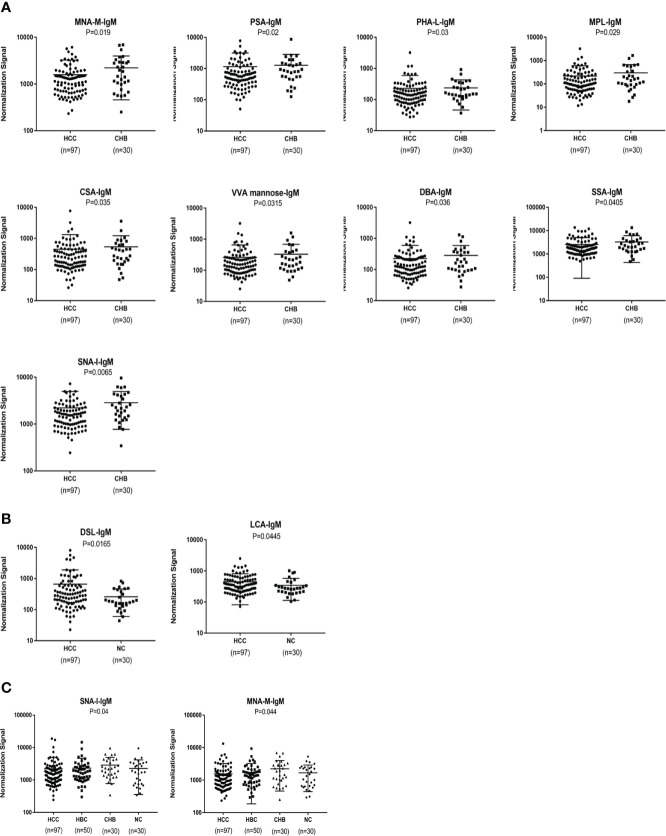
Specific lectins of serum IgM determined by the lectin microarray. **(A)** Specific differences in serum IgM glycosylation between HCC and CHB. **(B)** Specific differences in serum IgM glycosylation between HCC and NC. **(C)** Specific differences in serum IgM glycosylation among HCC, CHB, HBC, and NC.

An analysis of serum IgM glycosylation revealed that MNA-M, PSA, PHA-L, MPL, CSA,VVA, DBA, SSA and SNA-I signal intensities are lower in HCC than in CHB (P < 0.05) (**Figure 4A**). DSL and LCA had higher signal intensities in patients with HCC than in the NC group (P < 0.05) (**Figure 4B**). HBC, CHB, and NC were taken as a control group for subsequent analyses, and two differential lectins (SNA-I and MNA-M) with high IgM glycosylation levels in the HCC group were found (P < 0.05) (**Figure 4C**).

### Construction of a diagnostic model for HCC

We used binary logistic regression analysis of differentially expressed lectins and AFP to develop a diagnostic model by combining the relative signal values of lectin probes and AFP values in the training group of experimental samples. To verify the clinical value of the combined diagnostic model for HCC, an ROC curve analysis of the diagnostic efficacy was performed with the training group and validation group. The ROC curve analysis revealed that the combined model had good diagnostic ability.

**Figure 5:**
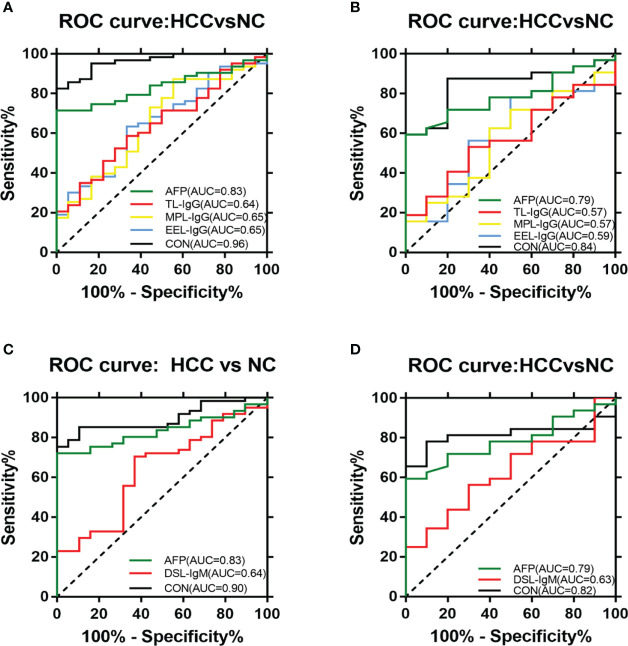
ROC analyses of the diagnostic value for HCC. **(A)** ROC analyses based on the IgG training group. **(B)** ROC analyses based on the IgG validation group. **(C)** ROC analyses based on the IgM training group. **(D)** ROC analyses based on the IgM validation group.

In terms of IgG, the combined diagnostic model based on AFP and lectins distinguished between HCC and NC in the training group, as determined by a binary logistic regression analysis. The effectiveness of the diagnostic model was significantly better than the effectiveness of AFP in distinguishing between HCC and NC (**Table 2**). The combination of three lectins and AFP significantly improved the diagnostic accuracy of HCC. The area under the curve was 0.96 (P < 0.05), sensitivity was 82.54%, while the specificity was 100% (**Table 4**) (**Figure 5A**).

**Table 2:** Results of the developed binary logistic regression model in HCC and NC of IgG.

						95% CI for Exp (B)	
Factor	Coefficient (B)	Standard error (SE)	Wals	P	Exp (B)	Lower	Upper
AFP	0.510	0.251	4.136	0.042	1.664	1.019	2.720
SBA	0.000	0.000	0.374	0.541	1.000	0.999	1.000
EEL	0.004	0.001	9.023	0.003	1.004	1.001	1.007
MPL	-0.008	0.003	5.640	0.018	0.992	0.986	0.999
TL	-0.015	0.007	4.432	0.035	0.985	0.972	0.999
Constant	-2.277	1.159	3.862	0.049	0.103		

The joint diagnostic model was as follows: -2.277 + 0.510*AFP + 0.004*EEL - 0.008*MPL - 0.015*TL

In terms of IgM, the newly developed predictive model was also significantly better than the effectiveness of AFP in distinguishing between HCC and NC (**Table 3**). The combination of one lectins and AFP significantly improved the diagnostic accuracy of HCC. When the one lectin were combined with AFP, the area under the curve was 0.90 (P < 0.05), sensitivity was 75.41%, and specificity was 100% (**Table 4**) (**Figure 5C**).

**Table 3:** Results of the developed binary logistic regression model in HCC and NC of IgM.

						95% CI for Exp (B)	
Factor	Coefficient (B)	Standard error (SE)	Wals	P	Exp (B)	Lower	Upper
AFP	0.286	0.129	4.944	0.026	1.331	1.034	1.712
DSL	0.003	0.001	3.983	0.046	1.003	1.000	1.005
LCA	-0.001	0.001	0.277	0.598	0.999	0.996	1.002
Constant	-1.901	0.873	4.743	0.029	0.149		

**Table 4:** Modeling evaluation of HCC group versus NC.

	Training group of IgG		validation group of IgG		Training group of IgM		validation group of IgM	
	Combined model	AFP (ng/ml)	Combined model	AFP (ng/ml)	Combined model	AFP (ng/ml)	Combined model	AFP (ng/ml)
Cut off value	2.07	20	2.07	20	2.07	20	2.07	20
Sensitivity(%)	82.54	53.97	78.13	59.38	75.41	53.97	65.63	59.38
Specificity(%)	100.00	100.00	80.00	100.00	100.00	100.00	100.00	100.00
AUC	0.96	0.83	0.84	0.79	0.9	0.83	0.82	0.79

The joint diagnostic model was as follows: -1.901 + 0.003*DSL + 0.286*AFP

Given the effectiveness of the joint diagnostic models to distinguish between HCC and NC for both IgG and IgM groups, we further evaluated a model by combining differential lectins for IgG and IgM. We found that the differential lectins identified by binary logistic regression were the same as those in the single IgG group; accordingly, the modeling was not repeated. An analysis of the validation group further supported this conclusion (**Figures 5B, D**).

## Discussion

The diagnosis of HCC is still very difficult, especially in the early stages of disease development, despite its importance for improving prognosis. However, no single biomarker with sufficient sensitivity and specificity for HCC detection has been reported to date. Methods to evaluate and detect the risk of HCC at an early stage are urgently needed. This is imperative for further in-depth studies investigating the molecular mechanism underlying the occurrence and development of HCC and the exploration of new targets for prevention and treatment. Combinations of multiple biomarkers have been shown to improve the early diagnosis.

Lectin microarrays are an emerging technique, first introduced in 2005 (26, 27). Unlike common polysaccharide analysis methods, lectin microarrays enable high-throughput, high sensitivity, and rapid profiling of complex glycan features to comprehensively characterize glycosylation directly (28, 29). These microarrays have been widely used in many recent studies of biological processes and diseases, such as cancer biomarker identification (30–32). In the present study, we evaluated 207 serum samples by a lectin microarray approach, including 97 cases of HCC, 50 cases of HBC, 30 cases of CHB, and 30 cases of NC. Analysis of serum IgG glycosylation revealed a difference in PHA-L signal intensities between HBC and HCC (P < 0.05). In an analysis of serum IgG glycosylation in patients with CHB and HCC, SBA had a higher signal intensity and the signal intensities of TL, IAA, PHA-E, AIA, VVA, and Black bean crude were significantly reduced. Compared with NC, SBA and EEL had higher signal intensities and MPL and TL had lower signal intensities in HCC (P < 0.05). By merging HBC, CHB, and NC into a control group, we identified SBA and ECL as differential lectins with high IgG glycosylation levels in the HCC group (P < 0.05), and AIA and MPL as differential lectins with low IgG glycosylation levels in the HCC group (P < 0.05). Serum IgM glycosylation analysis demonstrated that MNA-M, PSA, MPL, PHA-L, VVA, CSA, DBA, SSA and SNA-I show lower signal intensities in HCC than in CHB (P < 0.05). Additionally, DSL and LCA signal intensities were higher in patients with HCC than in NC (P < 0.05). Pooling HBC, CHB, and NC as the control group, we further identified SNA-I and MNA-M as differential lectins with high IgM glycosylation levels in HCC (P < 0.05).

IgG antibodies are glycoproteins, and each protein molecule contains an average of 2.8 N-linked glycans. Two N-linked glycans are always located at Asn297 in the Fc region of the two heavy chains, and additional N-linked glycans can be found in the Fab region (33). The two N-linked glycans in the Fc region have crucial functions in the structure and Fc-mediated biological function of IgGs (34). Therefore, exploring the glycosylation pattern and IgG levels in the serum of patients with HCC may contribute to a more comprehensive understanding of the pathogenesis of HCC.

In our study, we found a decrease in the galactosylation (Galβ4GlcNAc and Galβ3GlcNAc) of IgG in HCC. This finding was consistent with results for other diseases. For instance, the galactosylation of IgG in patients with rheumatoid arthritis is related to the severity and duration of illness, and the galactose ratio in patients with rheumatoid arthritis is significantly lower than that in healthy controls (P < 0.01) (35). Mehta et al. (36) found that compared to patients with mild fibrosis, patients with severe cirrhosis or fibrosis had greater alterations in the glycosylation of alpha-Gal IgG.

Studies have shown that sialylation process is related to innate anti-inflammatory activity (37, 38); however, the underlying mechanism is not clear. Sialylation of IgG antibodies can contribute to the regulation of the production of TH2 cytokines by innate myeloid cells in a DC-SIGN-dependent manner as well as the maintenance of homeostasis. This phenomenon could be regarded as an promising therapeutic target to inhibit the inflammation of autoimmune diseases (39). However, other studies have shown that sialylation only slightly changes the Fc domain structure and has little effect on DC-SIGN binding (40, 41). Therefore, the effects and clinical value of sialylation are presently unclear. However, we did not detect a significant difference in sialylation patterns between the HCC group and other groups, and this aspect should be studied further.

A lack of fucose can improve antibody-dependent cellular toxicity by enhancing binding to FcγRIIIa (42). This binding alleviates spatial conflict, stabilizes binding, and improves antibody affinity (43). In our study, we found that the fucosylation of serum IgG in patients with HCC was lower than that in patients with HBV and NC. This finding is not consistent with previous results. For instance, Saldova et al. (44) found increase in core fucosylation levels in advanced ovarian cancer. For HCV-infected individuals with fibrosis and cirrhosis, increased reactivity with several fucose-binding lectins has been reported (36). Further studies are needed to explain this discrepancy in the results.

IgM is the first antibody produced during immune response, the first antibody in the process of ontogeny, and the oldest antibody. IgM is the only antibody shared among all vertebrate species. N-linked glycosylation sites of IgM include Asn563 (tailpiece), Asn402 (Cμ3), Asn395 (Cμ3), Asn332 (Cμ2), and Asn171 (Cμ1) (45, 46). Colucci et al. (47) found that sialylated N-glycans play a key role in inducing IgM-mediated immune suppression. In our study, we found that sialylated N-glycans of serum IgM are reduced in HCC. Therefore, we speculate that changes in serum IgM glycosylation may be related to the occurrence of HCC.

AFP was used as a serological marker for diagnosing HCC as early as the 1960s and is currently the most widely used biomarker for HCC worldwide (48). However, the sensitivity value of AFP for the diagnosis of HCC is about 70%,and the specificity is still insufficient (49). Therefore, the American Association for the Study of Liver Diseases (AASLD) Practice Guidelines Committee does not recommend AFP as the only means of early detection of HCC (50). Because of the advantages of convenient and inexpensive serum markers for diagnosing liver cancer, It is hoped that new serum markers for liver cancer can be found to compensate for the shortage of AFP. By constructing a joint diagnostic model of differential lectins and AFP for distinguishing between patients with HCC and healthy individuals, the combination of lectins and AFP significantly improved the diagnostic accuracy of HCC over that of AFP alone. In terms of IgG, the area under the ROC curve was 0.96 (P < 0.05), the sensitivity was 82.54%, and the specificity was 100%. And in terms of IgM, the area under the curve of 0.90 (P < 0.05), sensitivity of 75.41%, and specificity of 100%.This result was subsequently validated in the validation group. It was further confirmed that the combined diagnostic model had higher diagnostic efficacy compared with AFP. The sensitivity of the combined diagnostic model was particularly high compared with that of AFP. Therefore, this study provides a feasible model for the diagnosis of HCC, especially for AFP-negative patients, and may serve as a powerful complement to *in vitro* diagnosis. Therefore, this study provides a feasible model for diagnosing HCC, especially in AFP-negative patients. The model can be used as a powerful supplement for in vitro diagnosis.

In our research, we added the HBC and CHB groups to establish effective combined diagnostic models that distinguish the HCC group from high-risk populations such as CHB and HBC. However, no models were found to improve diagnostic efficacy significantly. Although no valid model was established, we found differentially expressed lectins in HCC and CHB, HCC and HBC, and HCC and non-HCC, which we believe will help us in future studies on the pathogenesis of HCC.

There are also some limitations in our research. Due to the small sample size, it is still necessary to expand the sample size to eliminate systematic errors, so as to obtain highly reliable and determined markers. In addition, long-term follow-up studies are still needed to study the impact of changes in glycosylation on the survival of patients with HCC.

Our research is the first to apply lectin microarrays for analyzing changes in serum IgG and IgM glycosylation patterns in patients with HCC. The newly established joint diagnostic model could be an effective supplement to already established diagnostic biomarkers for HCC, providing a novel direction for further studies investigating the pathogenesis of HCC and biomarker identification.

## Data availability statement

The original contributions presented in the study are included in the article/**Supplementary Material**. Further inquiries can be directed to the corresponding author.

## Ethics statement

The studies involving human participants were reviewed and approved by the Ethics Committee of Shengjing Hospital of China Medical University. Written informed consent for participation was not required for this study in accordance with the national legislation and the institutional requirements.

## Author contributions

YZ, SZ and XQ performed the majority of the work described in this study. SZ performed the data analysis. YZ and XQ wrote and edited the manuscript. XQ supervised the project. All authors contributed to the article and approved the submitted version.

## Funding

This study was supported by Liaoning Provincial Central Government’s special project to guide local scientific and technological development (2019 JH6/10400009),the National Science and Technology Major Project of China (2018ZX10302205), the “345 Talent Project” of Shengjing Hospital of China Medical University.

## Conflict of interest

The authors declare that the research was conducted in the absence of any commercial or financial relationships that could be construed as a potential conflict of interest.

## Publisher’s note

All claims expressed in this article are solely those of the authors and do not necessarily represent those of their affiliated organizations, or those of the publisher, the editors and the reviewers. Any product that may be evaluated in this article, or claim that may be made by its manufacturer, is not guaranteed or endorsed by the publisher.
